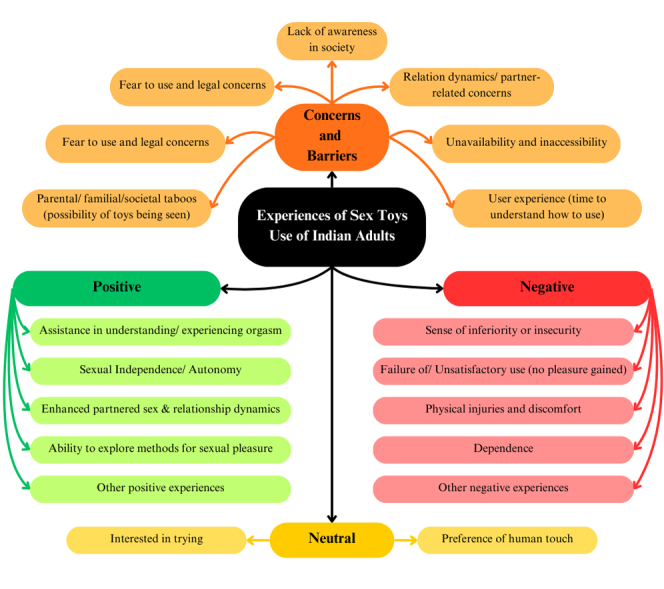# Exploring sex toy use among 18–45-year-old Indian adults: insights from a nationwide online survey

**DOI:** 10.1016/j.lansea.2024.100508

**Published:** 2024-11-11

**Authors:** Pauras Mhatre, Antara Agrawal, Pratham Agrawal, Gaurang Narayan, Priyanka Kataria, Pragati Rathod, Sarita Wadhva

**Affiliations:** a Seth G.S. Medical College and K.E.M. Hospital - GUG Office, Ground Floor, College and Administrative Office, Seth G.S. Medical College and K.E.M. Hospital, Acharya Donde Marg, Parel, Mumbai, Maharashtra, 400012, India; b Department of Community Medicine, Government Medical College and Hospital, Nagpur - Ground Floor, College Building, Government Medical College and Hospital - Nagpur, Hanuman Nagar, Nagpur, Maharashtra, 440003, India; c Department of Obstetrics and Gynaecology, Indira Gandhi Government Medical College & Hospital, Central Avenue, Mominpura, Nagpur, Maharashtra, 440018, India

## Comment

### Discussion

Sex toys, objects designed to induce or heighten sexual arousal, constitute a rich historical lineage of dildos and phallus-shaped objects dating back to the 3rd century in various civilizations.^1^, ^2^, ^3^ These devices play a crucial role in enhancing sexual pleasure, facilitating orgasms, as well as addressing medical conditions such as orgasmic dysfunctions following cancer treatment.

Despite the widespread incorporation of sex toys in sexual well-being, biopsychosocial contexts, and medical contexts, there is a noticeable dearth of scientific data in this regard. Cultural attitudes significantly shape the discourse around this subject, often dictating the level of openness and acceptance within different communities.^4^, ^5^, ^6^, ^7^ We used an online questionnaire-to estimate the extent of sex toy use in Indian adults, explore user experiences, analyse the relationship between sex toy use and demographic factors, and examine the correlation between sex toy use and general sexual satisfaction.

Remarkably, 98·5% of the 2071, 18–44 year old adults acknowledged awareness of sex toys, indicating a heightened openness to sexual exploration and pleasure in Indian society despite conservative cultural norms. This was also observed through the reported use of sex toys by 40·56% of participants in both solo and partnered sex. Indian adults who were 25–44 years old were about twice as likely to use sex toys than those aged 18–24 years. Usage of sex toys was significantly higher in cisgender women, non-cisgender individuals, and people identifying as non-heterosexual/queer. While a significant number of participants shared positive experiences, highlighting the role of sex toys in understanding orgasms, promoting sexual arousal, and enhancing pleasure, some participants also expressed negative experiences such as feelings of inferiority, insecurities, and physical health issues. Major concerns primarily revolved around social taboos, judgments, and challenges related to privacy, accessibility, and awareness. These findings underscore the diverse range of perspectives on sex toys, emphasizing the importance of addressing social stigmas and fostering awareness. The detailed methods and results are provided for readers’ interest in the Appendix.

Recently, India has started making definitive strides in the direction of sexual literacy as reflected by the rising number of sex educators and sexual wellness influencers and the growing market for sex toys. Sexual wellness influencers, in addition to offering a safe space to discuss intimacy, help rectify individuals' perception of sex toys - from products addressing the ‘orgasm gap’ for women to products that promote and support safe, consensual, and playful sex for all genders and sexualities. Healthcare professionals can facilitate and expedite this change by not just including sexual wellness devices in comprehensive treatment approaches for sexual dysfunctions but also by educating patients and creating a space for open discussion on the use of sex toys.

While society is gradually embracing new individualized moralities, the legal system remains stuck in the past, often presenting obstacles rather than facilitating change. Currently, there is no explicit legal provision in India that bars the sale of sex toys. However, Section 292 of the Indian Penal Code (equivalent to Section 294 of the new Bharatiya Nyaya Sanhita) has been frequently invoked to restrict the sale, exhibition, advertising, import, or export of sex toys on the grounds of “obscenity.” The determination of what constitutes obscenity is subjective, often based on varying moral perspectives.^8^ This lack of judicial clarity exacerbates issues related to the importation and marketing of sex toys.

To navigate these legal complexities, companies often label sex toys as ‘massagers,’ a misrepresentation that raises concerns about consumer protection laws and increases the likelihood of import penalties for misclassification.^9^ These laws also affect the types of sex toys available to consumers, as most sellers resort to offering only vibrators, which can be disguised as everyday items like lipsticks or pens.^10^ This limited range of products systematically excludes certain sections of society, such as the LGBTQ community, adding to the social discrimination they face despite the abrogation of Article 377.

These challenges highlight the urgent need for legislative reforms, including the creation of a separate category for sex toys within the Harmonized System of Nomenclature (HSN) codes and the establishment of clear guidelines for their marketing and sale, ensuring that they are safe, free from harmful chemicals, and not detrimental consumers’ health. In conclusion, such public health and legal reforms can significantly enhance sexual health and promote a more inclusive and sex-positive society.

## Contributors

PM, PR, and SW conceptualized the study and were involved in its supervision and validation. PM and PA looked after the overall project administration. AA, PA, GN, PK, and PM contributed collectively to the data curation, methodology, and investigation. PM and GN carried out the formal analysis of the study data. All authors had full access to all the data in the study. PM, AA, and GN have directly accessed and verified the data reported in the manuscript. PM, AA, and GN wrote the original draft of the manuscript. PA, PR, SW, PK, and PM were involved in the review and provided inputs on various aspects of the manuscript. All authors reviewed and approved various drafts and the final paper.

## Data sharing statement

All of the de-identified individual participant/study data collected during the survey will be available upon request by emailing the corresponding author.

## Declaration of interests

All authors declare that they have no relevant or material financial or non-financial interests competing interests to report.

## Figures and Tables

**Supplementary Figure S1:**
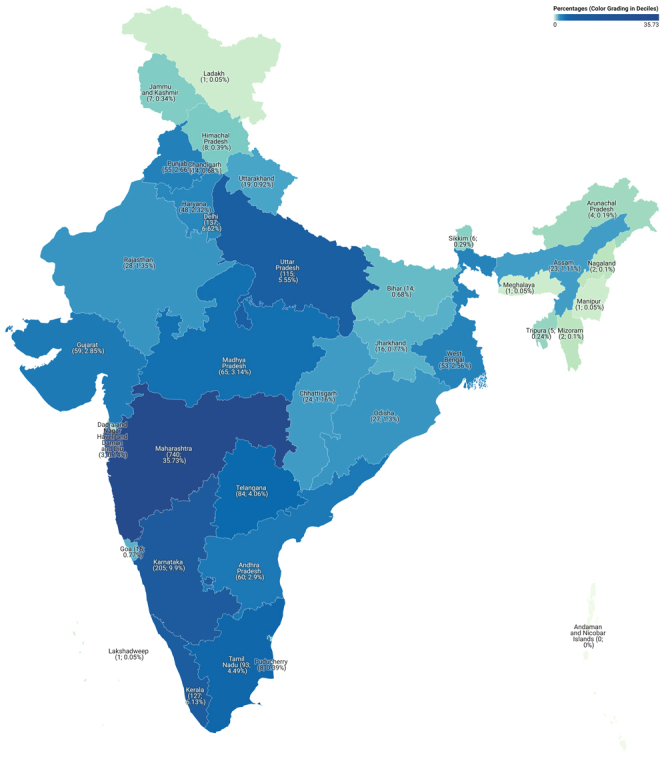


**Supplementary Figure S2:**
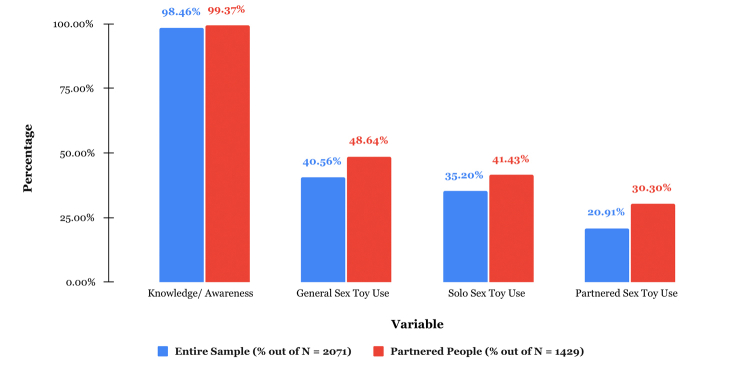


**Supplementary Figure S3:**
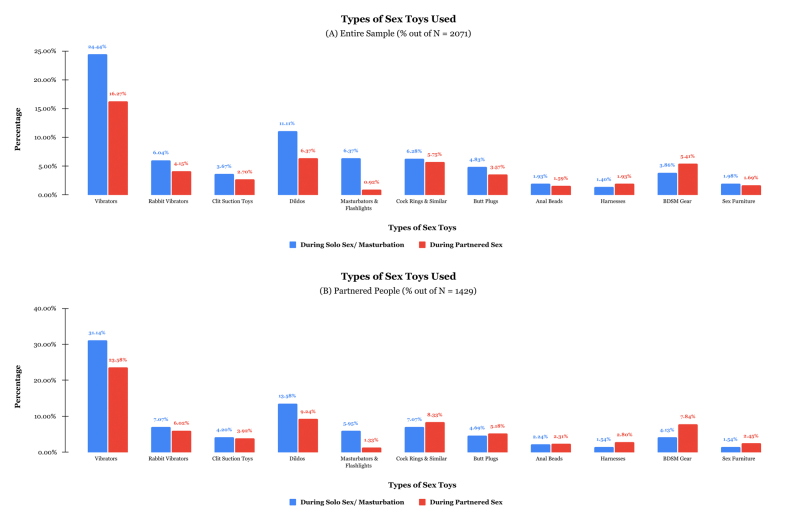


**Supplementary Figure S4:**
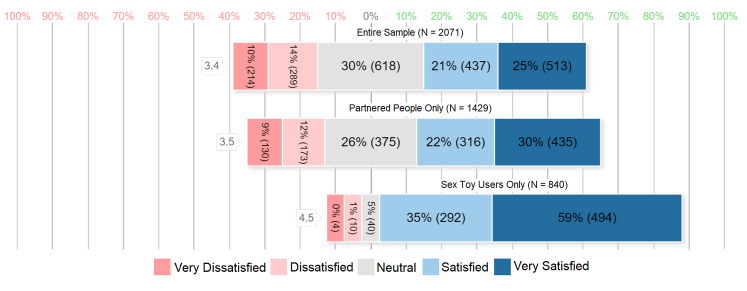


**Supplementary Figure S5:**