# Supplementary material for: Exploring sex toy use among 18–45-year-old Indian adults: insights from a nationwide online survey
Source: Lancet Reg Health Southeast Asia. 2024 Nov 11;32:100508. doi: 10.1016/j.lansea.2024.100508 (PMC11585771; doi:10.1016/j.lansea.2024.100508)
Supplement: Appendix [file mmc1.pdf]

Appendix: Detailed Methods and Results of the Study

Methods

Study Design, Setting & Duration

This cross-sectional online questionnaire-based study was conducted in all States and Union Territories (hereafter referred to as 'state') of India over a period of eight months from June 2022 to January 2023.

Ethical Considerations

Approval of the Institutional Ethics Committee (Approval Letter No. EC/Pharmac/GMC/NGP/3703 dated 30th May 2022) was sought before beginning the study. Written informed consent was obtained from the participants in the language they could comprehend using a consent form appended to the questionnaire (available in 12 different languages). The institutional ethics committee approved the use of electronically obtained consent, acknowledging the minimal risk associated with the study. No personal identifying information of the study participants was collected to ensure anonymity and confidentiality. The study was conducted according to the principles stated in the Declaration of Helsinki, 2013, ICH-GCP Guidelines, and the ICMR's Ethical Guidelines for Biomedical Research on Healthy Participants, 2011.

Study Sample

Sample Size: In the absence of available literature on the prevalence of sex toy use in India, we relied on international studies as a point of reference. In a study by Nicola Döring and Sandra Poeschl, it was found that 45% of German heterosexual adults had used sex toys during solo sex while 52% incorporated sex toys in partnered sexual activities.[1] For maximum variability, the higher 52% ($p = 0{\cdot}52$ and $q = 1 - p = 0{\cdot}48$) was employed for sample size calculation, maintaining a 95% confidence level ($Z = 1{\cdot}96$) and 5% precision (e). Using Cochran's formula ($n \geq Z^2pq/e^2$), the minimum required sample size, after rounding off, was determined to be 400 participants. However, given the nationwide scope of our study, we accepted the maximum number of possible responses above this minimum requirement throughout the study period.

Sampling: Non-probability, snowball-cum-voluntary response sampling technique was used for the study. Every participant meeting the following inclusion criteria were enrolled in the study: (i) individuals of any sex, gender, or sexual orientation identifying as Indian citizens; (ii) individuals aged between 18 and 44 years; (iii) individuals with the ability to comprehend and use electronic devices; and (iv) individuals who willingly consented to participate.

Study Tools and Outcome Measures

The study utilized a self-designed and pre-validated multilingual questionnaire (English version attached at the end), validated using a two-step process of - expert review ensuring its reliability (content validity ratio = 1·00, scale level content validity index = 1·00, Kuder-Richardson-20 Statistic for Internal Consistency = 0·844), followed by a pilot study in the second step carried out on 20 participants. The questionnaire was distributed nationwide through popular social media platforms like WhatsApp, Telegram, Instagram, etc., using Google Forms. Available in 12 languages (English, Hindi, Bengali, Marathi, Telugu, Tamil, Gujarati, Urdu, Kannada, Malayalam, Punjabi, and Assamese), the self-administered questionnaire aimed to maximize accessibility and representation. Participants provided electronic consent through an attached informed consent document appended to the Google Form.

The study questionnaire collected sociodemographic information about the state/union territory of residence, age group, sex, gender, sexual orientation, and history of current/past sexual partner(s). To be sensitive, inclusive, and affirmative about people of various gender identities and sexual orientations, the questions about gender and sexual orientation provided a comprehensive list of options of labels to choose from, including an option to type their preferred label (in case they preferred a label other than the provided options). Furthermore, the questionnaire collected information on the participants' knowledge and awareness of the existence of sex toys. The meaning of sex toys was subsequently explained, and further questions on the extent of sex toy use (and their types) and experience with sex toys were asked. A question on general sexual satisfaction was asked. These questions were multiple choice questions, either binary yes/no-type or Likert scale type. Finally, an optional open-ended question asked participants about their views, opinions, experiences, concerns, etc., about sex toys.

Statistical Analysis

The data collected via Google Forms were automatically compiled into an Excel spreadsheet. Subsequent statistical analysis was carried out using JASP software (The JASP Team, 2022). Descriptive statistics are presented as numbers (frequencies) and percentages. Various statistical tests, including the chi-square test, Kendall's tau test, and prevalence odds ratio (utilizing multivariate logistic regression), were employed to examine associations between variables. Associations with p values $< 0{\cdot}05$ were considered statistically significant.

Qualitative data obtained from open-ended questions underwent exploratory analysis using thematic analysis methods. Themes and subthemes were identified, and two team members (PM and GN) independently coded the data. Key responses related to specific themes and subthemes were quoted to enhance the clarity and dissemination of information.

Role of Funding Source

The authors received no funding for this study.

Results

The study included all 2071 individuals who responded to the online questionnaire after ensuring the completeness of their forms. Nonresponse or exclusion rates couldn't be reported due to a lack of precise quantitative data on the form's reach. Participants from across India participated (Figure 1), with the majority from Maharashtra (35·7%), followed by Karnataka (9·9%) and Delhi (6·6%).

Figure 1: States and union territories of study participants

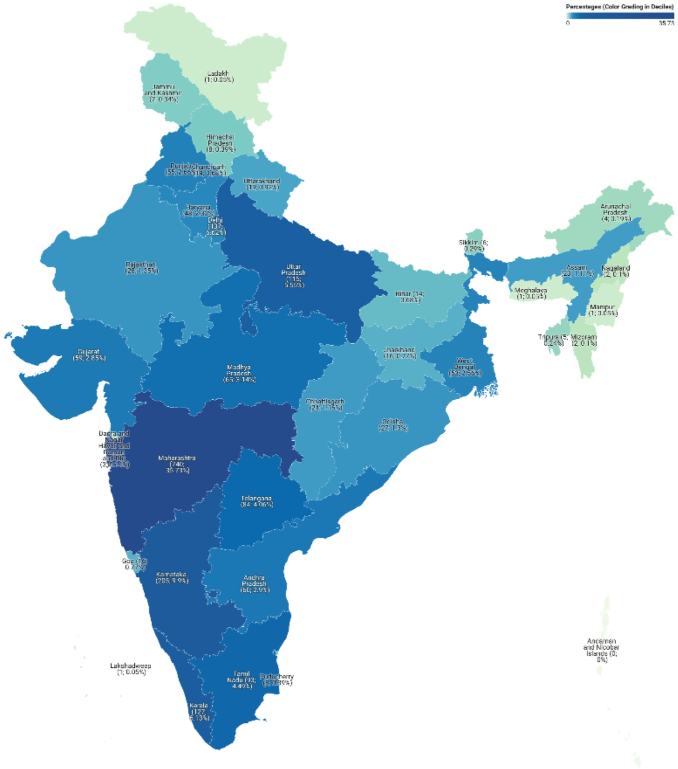

Sociodemographic Profile of the Study Sample

Table 1 presents a summary of the sociodemographic information of the study participants. The majority of participants belonged to the 18 to 24 age group. The study included almost an equal number of male and female participants. Likewise, there were nearly equal proportions of cisgender men and cisgender women, with a small percentage (3%) identifying as non-cisgender (transgender and gender-diverse populations). The majority of participants identified as heterosexual (85·4%) in terms of sexual orientation. Additionally, most participants reported having one or more sexual partner(s), either presently or in the past (69%).

Table 1: Sociodemographic information of study participants

**Variable**	**Frequency**	**Percentage (%)**
Total	2071	100
Age group		
18 – 24	1269	61·28
25 – 44	802	38·73
Sex		
Female	1032	49·83
Male	1039	50·17
Intersex	0	00·00
Gender		
Cisgender Man	1005	48·53
Cisgender Woman	1004	48·48

Non-Cisgender[a]	62	2·99
Sexual orientation		
Heterosexual	1768	85·37
Non-Heterosexual/ Queer Spectrum[b]	303	14·63
(History of) Sexual Partner(s) (Present and/or Past)		
Yes	1429	69·00
No	642	31·00

a. For the purpose of statistical analysis and simplicity of representation; Agender, Gender Fluid, Gender Non-binary, Gender Non-conforming, Genderqueer, No labels, Prefer not to say, Transgender Woman; were grouped together as 'Non-Cisgender'.
b. For the purpose of statistical analysis and simplicity of representation; Asexual (+ace spectrum), Autosexual, Bisexual (+ spectrum), Demisexual, Questioning, Gay (Homosexual man), Gynesexual, Homosexual, Lesbian (Homosexual woman), None, Pansexual, Prefer not to say, and Queer; were grouped together as 'Non-Heterosexual/ Queer Spectrum'.

Knowledge and Proportion of Use of Sex Toys Among Surveyed Participants

Figure 2 shows the percentage of the participants who knew about sex toys and/or used them. What sex toys are and what they are used for, was known to 98·5% of Indian young and early-middle-aged adults participating in the study. A total of 40·56% of Indian young and early-middle-aged adults participating in the study reported that they had used sex toys in general, that is, either in solo or partnered sex. A total of 35·2% of all the participants reportedly used sex toys specifically during solo sex/ masturbation. On the other hand, 30·3%, of the participants who had sexual partners, reported the use of sex toys specifically during partnered sex (including group sex).

Figure 2: Knowledge and proportion of use of sex toys among surveyed participants

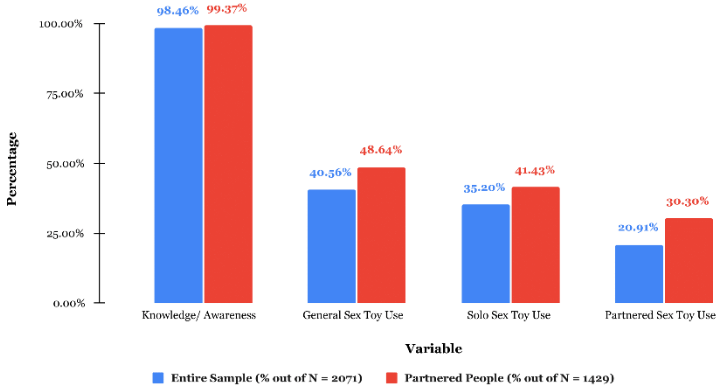

Figure 3 shows the types of sex toys used by our study sample. As reported, the most commonly used sex toys were vibrators for both types of sex, solo (24·4%) and partnered (16·3%). Dildos, rabbit vibrators, masturbators & flashlights, and cock rings were other types of sex toys that were reportedly used commonly.

Figure 3: Use of different types of sex toys (A) Entire sample (B) Partnered people

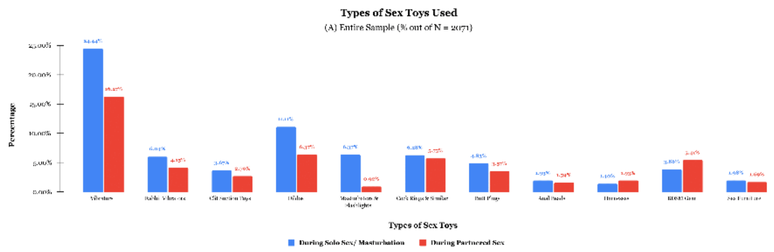

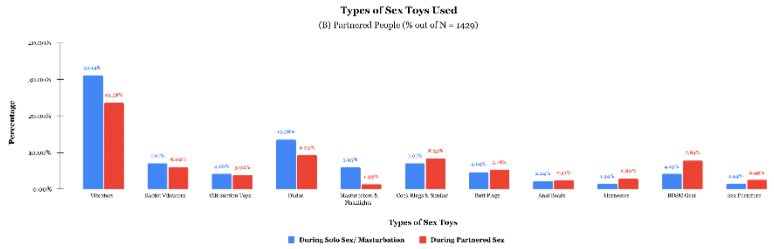

Association of Knowledge and Use of Sex Toys with Sociodemographic Variable

A statistical analysis was performed to find associations between sex toy use and demographic factors using the Chi-square test, and Prevalence Odds Ratio using multivariate logistic regression (Table 2). While no significant difference was found between the two age groups in terms of knowledge about the existence of sex toys, a notable discrepancy ($p < 0{\cdot}05$) emerged in the usage of sex toys (general, solo, and partnered), favouring the higher age group. Specifically, Indian adults aged 25 to 44 years exhibited a 2·04 times higher proportion of sex toy use compared to those aged 18 to 24 years.

Although sex was found to be associated with both the knowledge of the existence of sex toys and their usage (general, solo, and partnered) on the Chi-square test; this result was modified when adjusted for other variables on multivariate logistic regression where sex was associated only with solo sex toy use (male adults were 1·92 times more likely to use them in solo). Similar effects were seen in the analysis for gender, where after adjusting for confounders, cisgender women and non-cisgender people were more likely to use sex toys.

No significant distinction was observed between the broad categories of sexual orientation regarding knowledge of the existence of sex toys. However, a notable difference ($p < 0{\cdot}05$) emerged among different sexual orientations in terms of sex toy usage (general, solo, and partnered), with a higher proportion observed among queer (non-heterosexual) individuals, who were 2·09 times more likely to use sex toys. Additionally, a significant association was identified between the history of sexual partners (presently or in the past) and sex toy usage. Individuals who have had sexual partners were 2·46 times more likely to use sex toys.

Table 2: Association of sociodemographic variables with knowledge and use of sex toys

**Variable**	**Yes n (%)**	**No n (%)**	**Total n (%)**	**Chi-Square Value**	**p value**	**Adjusted Prevalence Odds Ratio[a] (95% CI)**	**p value**
**AGE GROUP**							
Knowledge							
18 – 24	1245 (60·12 %)	24 (1·16 %)	1269 (61·28 %)	2·58	0·108	(Reference)	-
25 – 44	794 (38·34 %)	8 (0·39 %)	802 (38·73 %)			1·163 (0·503 – 2·685)	0·724
General Sex Toy Use							
18 – 24	415 (20·04 %)	854 (41·24 %)	1269 (61·28 %)	83·912	< 0·001[b]	(Reference)	-
25 – 44	425 (20·52 %)	377 (18·20 %)	802 (38·73 %)			2·037 (1·677 – 2·475)	< 0·001[b]
Solo Sex Toy Use							
18 – 24	371 (17·91 %)	898 (43·36 %)	1269 (61·28 %)	51·113	< 0·001[b]	(Reference)	-
25 – 44	358 (17·29 %)	444 (21·44 %)	802 (38·73 %)			1·765 (1·445 – 2·156)	< 0·001[b]
Partnered Sex Toy Use							
18 – 24	166 (11·62 %)	594 (41·57 %)	760 (53·18 %)	54·999	< 0·001[b]	(Reference)	-

25 – 44	267 (18·68 %)	402 (28·13 %)	669 (46·82 %)			2·51 (1·977 – 3·187)	< 0·001[b]
**SEX**							
Knowledge							
Male	1013 (48·91 %)	26 (1·26 %)	1039 (50·17 %)	12·559	< 0·001[b]	(Reference)	-
Female	1026 (49·54 %)	6 (0·29 %)	1032 (49·83 %)			0·359 (0 – 1013·01)	0·801
General Sex Toy Use							
Male	332 (16·03 %)	707 (34·14 %)	1039 (50·17 %)	64·058	< 0·001[b]	(Reference)	-
Female	508 (24·53 %)	524 (25·30 %)	1032 (49·83 %)			0·31 (0·126 – 0·76)	0·011[b]
Solo Sex Toy Use							
Male	254 (12·27 %)	785 (37·90 %)	1039 (50·17 %)	105·711	< 0·001[b]	(Reference)	-
Female	475 (22·94 %)	557 (26·90 %)	1032 (49·83 %)			0·52 (0·231 – 1·169)	< 0·001[b]
Partnered Sex Toy Use							
Male	156 (10·92 %)	473 (33·10 %)	629 (44·02 %)	16·091	< 0·001[b]	(Reference)	-
Female	277 (19·38 %)	523 (36·60 %)	800 (55·98 %)			0·785 (0·296 – 2·079)	0·626
**GENDER**							
Knowledge							
Cisgender Man	979 (47·27 %)	26 (1·26 %)	1005 (48·53 %)	14·07	< 0·001[b]	(Reference)	-
Cisgender Woman	998 (48·19 %)	6 (0·29 %)	1004 (48·48 %)			9·05 (0·003 – 25418·457)	0·587
Non-Cisgender	62 (2·99 %)	0 (0·00 %)	62 (2·99 %)			$4·550\times10^{+6}$ (0 – ∞)	0·985
General Sex Toy Use							
Cisgender Man	305 (14·73 %)	700 (33·80 %)	1005 (48·53 %)	91·393	< 0·001[b]	(Reference)	-
Cisgender Woman	494 (23·85 %)	510 (24·63 %)	1004 (48·48 %)			5·975 (2·411 – 14·808)	< 0·001[b]
Non-Cisgender	41 (1·98 %)	21 (1·01 %)	62 (2·99 %)			7·114 (2·954 – 17·131)	< 0·001[b]
Solo Sex Toy Use							
Cisgender Man	231 (11·15 %)	774 (37·37 %)	1005 (48·53 %)	131·442	< 0·001[b]	(Reference)	-
Cisgender Woman	462 (22·31 %)	542 (26·17 %)	1004 (48·48 %)			4·828 (2·119 – 10·998)	0·114
Non-Cisgender	36 (1·74 %)	26 (1·26 %)	62 (2·99 %)			4·582 (2·087 – 10·06)	< 0·001[b]
Partnered Sex Toy Use							
Cisgender Man	148 (10·36 %)	458 (32·05 %)	606 (42·41 %)	17·623	< 0·001[b]	(Reference)	-
Cisgender Woman	271 (18·96 %)	506 (35·41 %)	777 (54·37 %)			2·009 (0·744 – 5·423)	0·168
Non-Cisgender	14 (0·98 %)	32 (2·24 %)	46 (3·22 %)			1·273 (0·502 – 3·224)	0·611
**SEXUAL ORIENTATION**							
Knowledge							
Heterosexual	1739 (83·97 %)	29 (1·40 %)	1768 (85·37 %)	0·719	0·397	(Reference)	-
Non-Heterosexual/ Queer Spectrum	300 (14·49 %)	3 (0·15 %)	303 (14·63 %)			1·078 (0·321 – 3·625)	0·903
General Sex Toy Use							
Heterosexual	663 (32·01 %)	1105 (53·36 %)	1768 (85·37 %)	46·937	< 0·001[b]	(Reference)	-

Non-Heterosexual/ Queer Spectrum	177 (8·55 %)	126 (6·08 %)	303 (14·63 %)			2·087 (1·594 – 2·732)	< 0·001[b]
Solo Sex Toy Use							
Heterosexual	561 (27·09 %)	1207 (58·28 %)	1768 (85·37 %)	63·777	< 0·001[b]	(Reference)	-
Non-Heterosexual/ Queer Spectrum	168 (8·11 %)	135 (6·52 %)	303 (14·63 %)			2·496 (1·906 – 3·268)	< 0·001[b]
Partnered Sex Toy Use							
Heterosexual	339 (23·72 %)	854 (59·76 %)	1193 (83·49 %)	12·155	< 0·001[b]	(Reference)	-
Non-Heterosexual/ Queer Spectrum	94 (6·58 %)	142 (9·94 %)	236 (16·52 %)			2·002 (1·465 – 2·737)	< 0·001[b]
**HISTORY OF SEXUAL PARTNERS**							
Knowledge							
No (Did not have/had partners)	619 (29·89 %)	23 (1·11 %)	642 (31·00 %)	25·388	< 0·001[b]	(Reference)	-
Yes (Have/had partners)	1420 (68·57 %)	9 (0·44 %)	1429 (69·00 %)			4·595 (2·038 – 10·357)	< 0·001[b]
General Sex Toy Use							
No (Did not have/had partners)	145 (7·00 %)	497 (24·00 %)	642 (31·00 %)	124·686	< 0·001[b]	(Reference)	-
Yes (Have/had partners)	695 (33·56 %)	734 (35·44 %)	1429 (69·00 %)			2·457 (1·962 – 3·077)	< 0·001[b]
Solo Sex Toy Use							
No (Did not have/had partners)	137 (6·62 %)	505 (24·38 %)	642 (31·00 %)	78·368	< 0·001[b]	(Reference)	-
Yes (Have/had partners)	592 (28·59 %)	837 (40·42 %)	1429 (69·00 %)			1·905 (1·511 – 2·401)	< 0·001[b]

a. Calculated using multivariate logistic regression
b. p value < 0·05 was considered statistically significant

Experience of Sex Toys and Comparison of General Sexual Satisfaction

Figure 4 illustrates the reported sexual satisfaction levels of all participants. Notably, the distribution of reported sexual satisfaction among the entire sample and partnered individuals leaned slightly toward the positive side. However, sex toy users showed a significant shift towards higher levels of satisfaction. As seen in Table 3, sex toy use was found to be significantly associated ($p < 0{\cdot}05$) with increased sexual satisfaction. Regarding responses to a binary yes-or-no question about feelings of jealousy or insecurity if their partner expressed a desire to use sex toys, 96·6% of partnered individuals (N = 1429) indicated that they did not or would not experience such feelings.

Figure 4: General sexual satisfaction of study participants

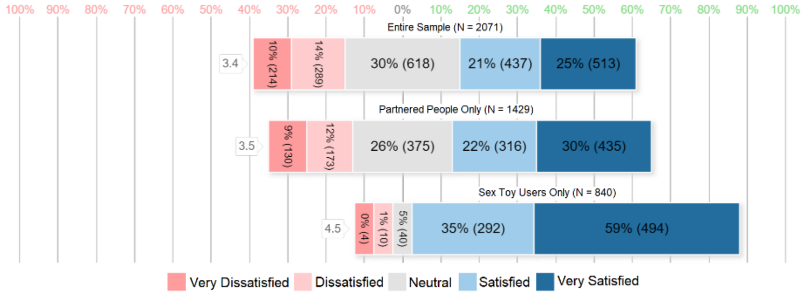

Table 3: Association of sex toy use with general sexual satisfaction

General Sexual Satisfaction	Sex Toy User n (%)	Sex Toy Non-User n (%)	Total n (%)	Chi-Square Value	p value	Kendall's Tau-b	p value
Very Dissatisfied	4 (0·19 %)	210 (10·14 %)	214 (10·33 %)	1381·734	< 0·001 [a]	34·964	< 0·001 [a]
Dissatisfied	10 (0·48 %)	279 (13·47 %)	289 (13·96 %)				
Neutral	40 (1·93 %)	578 (27·91 %)	618 (29·84 %)				
Satisfied	292 (14·10 %)	145 (7·00 %)	437 (21·10 %)				

Very Satisfied	494 (23·85 %)	19 (0·92 %)	513 (24·77 %)				
Total	840 (40·56 %)	1231 (59·44 %)	2071 (100·00 %)				

a. p value < 0·05 was considered statistically significant

Qualitative Analysis of Experience and Opinions About Sex Toys

A total of 188 participants responded to the optional open-ended question asking about their views, opinions, experiences, concerns, etc. about sex toys. As the open-ended question was not based on a structured/semi-structured guide, responses have not been reported in percentages. Four main themes were explored (Figure 5) through the responses of participants viz. 'Positive Experiences and Opinions', 'Negative Experiences and Opinions', 'Neutral Experiences and Opinions', and 'Concerns'.

Figure 5: Concept Map of Thematic Analysis of Qualitative Responses

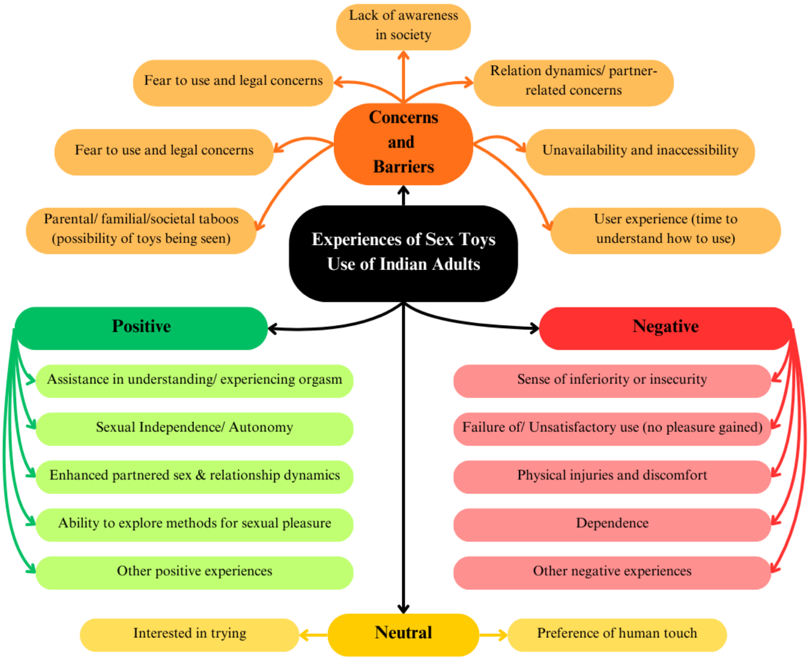

Positive Experiences and Opinions:

Close to 58 participants mentioned that sex toys helped them understand and experience orgasms. A middle-aged asexual cisgender woman shared how sex toys positively impacted her sexual experiences, stating, "I'm a sex-positive asexual. So, they have gone a long way in sexual arousal and/or pleasing my partner without penetrative sex". Whereas, a pansexual cisgender middle-aged woman (18–24 years) also expressed positive experiences with household items, highlighting their pleasant experiences and increased excitement and pleasure. A cisgender bisexual woman (18–24 years) noted that sex toys, especially in monogamous relationships, stimulate multiple erogenous zones, enhancing pleasure and providing convenience when feeling lazy to masturbate. Another participant emphasized the fun involved in sexual activities with sex toys, mentioning the addition of components such as lube and listening to erotica.

46 participants shared experiences of feeling independence/ autonomy and confidence through sex toy use. A young heterosexual cisgender woman (18–24 years) emphasized the "lifesaving" aspect of sex toys for those without a sexual partner, providing pleasure without dependence, and overcoming issues related to sexual intercourse. Another heterosexual cisgender woman stated, "Having a toy handy during solo sessions allowed me to explore my sexuality at my own pace, in privacy, and discover what works for my body. It helped build sexual confidence and awareness and helped communicate my needs to my partner". Another participant expressed that using sex toys made them feel more confident and powerful, freeing them from dependence on another person for sexual satisfaction.

27 participants reported having had better sexual experiences with partnered sex due to sex toy use. A young asexual gender-fluid female participant shared how sex toys helped explore their bodies with a partner, building a better sex life and promoting comfort in their bodies. An asexual, middle-aged, gender-nonconforming female participant explained the importance of sex toys for sexually diverse people by stating, "I come under the ace spectrum, more specifically, demisexual. I think using sex toys will be more exploratory between partners who want to be more intimate and find different ways of pleasure, instead of just the traditional form of penetrative sex in which women mostly are not considered for [experiencing] orgasm". One participant added that sex toys helped their partner understand their pleasure preferences, mimicking the speeds and variations with their fingers, and contributed to a richer sexual experience. Furthermore, a heterosexual cisgender man (25–44 years) shared, "Me & my wife both

love sex toys and use them liberally. They enrich our experience and keep sex interesting. I hope India will lose the stigma around sex toys. It doesn't look likely though".

A bisexual cis-gender woman described her experiences with vibrators as an ally, providing convenience and intimacy and allowing her to focus on herself or her partner, emphasizing the addition of toys as a fun element. Other participants shared experiences of sex toys opening avenues in relationships, adding variety, enhancing foreplay, and aiding in preparation for penetration. Finally, ten participants expressed a general liking for the use of sex toys.

Negative Experiences and Opinions:

Four participants expressed how the use of sex toys created a sense of inferiority or insecurity in themselves. Five participants expressed a sense of discomfort about using sex toys. A young heterosexual cis-gender man highlighted how it creates a sense of inferiority in males. Another person with similar demographics shared, "[I] have never tried using sex toys in partnered sex but sometimes feel that using sex toys might lead to no requirement of partnered sex. You may call it insecurity or whatever". A middle-aged heterosexual cisgender woman acknowledged the dip in confidence used by sex toys but suggested healthy conversations with partners to resolve such issues. A bisexual young cisgender man shared that he doubted his sexuality just because he did not want to use sex toys. He shared, "I was encouraged to use a dildo once but I refrained from it because the circumstances it was given in were just not right, and [it was] not safe. In addition, that kind of made me a little sceptical about using bisexual sex toys or even admitting that I am bisexual or not". Some other key responses in this context included that sex toys looked bad in advertisements below pornographic videos, etc.

Three participants expressed the belief that sex toys were not useful in enhancing sexual experiences. A young asexual cis-gender woman shared how the inability to orgasm with sex toys affected her self-esteem. From a different angle, one more young heterosexual sex participant added the limitations of sex toys, noting their focus on specific body parts and the absence of touch and feel sensations on other parts.

Six participants reported adverse physical health effects from using sex toys. A heterosexual middle-aged cis-gender woman described initial discomfort and fear of electric shocks while using a vibrator, recounting a specific incident with resistance bands (used by her partner on her for bondage) causing numbness in her hands as the band was tied too tight. Other reported issues include pain, unusual sensations while one's hand held the vibrator for a long time, urine passing due to pressure on the bladder, abrasion (if lubrication was not used), and the toy being stuck in the anus.

Lastly, four participants mentioned difficulty achieving orgasm without sex toys. A heterosexual cisgender woman expressed, "Sex toys make things easier (orgasm to be achieved) but also increase your threshold massively [the word 'massively' stressed using capital letters]. [I] have trouble with dependency on them as [I] cannot achieve orgasm easily without them, especially when I was off sex for a long time and only had them to rely on".

Neutral Experiences and Opinions:

Thirty-two participants shared that although they had not tried to use sex toys at the time of their response, they would like to try using them in the future. However, nine respondents expressed that they (would) prefer sexual experiences/exploration without sex toys or that they prefer human interaction/their partner. A questioning female person (who preferred no labels for her gender identity) shared, "*After the thrill of a new thing wore off, I found that using my hands was still better than my vibrator*".

Concerns Related to Sex Toys:

The common concerns raised by the participants (14 responses) included parental and social taboos, fear of judgment, and the challenge of 'getting caught'. A heterosexual young cisgender woman (18–24 years) elaborated in this context, "*I have never had a sex toy before, yet I want to get one. As I am working from home, I do not have the privilege of getting a sex toy and having it in my home without getting caught, even though I have room for myself. My parents are not interrogative; they never checked my room, yet I do not feel safe/comfortable having a sex toy around them. Even if some young adults think that using a sex toy is a sin, how would our parents who are from different generational backgrounds come to understand it? However, if I get to work offline, I would live away from my parents, where I could have a sex toy in my own room. This is not just me, even my friends feel the exact same thing!*".

Additionally, common obstacles cited while acquiring sex toys were unavailability and difficulty in access (22 responses), expensive/ affordability concerns (13 responses), lack of awareness in society (12 responses), and fear of use (including legal concerns) (8 responses). A pansexual cisgender woman (18–24 years) shared, "[I] *always wanted to use sex toys but couldn't find any affordable ones in India due to its ban and also due to the high prices that can't fit in a budget of a college student who is still unemployed*". Another heterosexual cisgender woman stated, "*I'm keen on using one but* [I] *don't know where to buy one. The only thing that the A-Z marketplace doesn't sell that widely in India*". Some other responses related to lack of awareness were [excerpts], "*Sex toys should be easily made available in Indian markets and more awareness should be created regarding their use.*" and "*I feel guilty because we are taught as women it's wrong to express ourselves sexually. And also, there is this whole set of myths saying you will never be satisfied by an actual person if you use sex toys.*"

Concerns surrounding partners and relationships were mentioned by 16 participants. A heterosexual middle-aged cisgender woman shared her narrative story, which highlights the problems related to the use of sex toys in a relationship. She described, "*I had no feelings of discomfort/jealousy when my partner (a cisgender male) and I (a cisgender female) used sex toys, and I even liked the experience as we had a more comfortable experience during intercourse after using the sex toy. We had both talked and discussed about using it beforehand, and I had bought the sex toy on his recommendation. I mentioned that I had a more comfortable experience with the sex toy because I suffer from a condition called the Fourchette tear most of the time during sexual intercourse. The vibrator and dildo helped me with natural lubrication and made the penetration experience easier and smoother for both of us. However, a little while later, my partner did experience feelings of jealousy and, more specifically, inadequacy and inferiority complex because I derived so much pleasure from a sex toy. In his words, he asked me, 'How could an inanimate piece of silicone*

be more pleasurable to you than my penis? I feel less of a man now since I know that I cannot satisfy you.'". Emphasizing the need for awareness and sexist taboos surrounding the use of sex toys by women, one participant added, "*There is a lot of stigmas in men if their female partner shows interest in using sex toys. Their male ego is extremely little, and they get offended. My personal experience with sex toys for masturbation has been amazing, and I feel there should be awareness regarding the same, especially for men; they should be comfortable with their sexual masculinity while having sex".* Other key responses to be noted by heterosexual cisgender women were *"My partner kind of had an issue with me using a vibrator regularly but I needed it because we are in a long-distance relationship. He thought it might affect our sex life but honestly, when I met him, it hardly made any difference";* and *"I haven't used any sex toys before. But in partner sex, I want to use vibrators. But in India, we (females) cannot convey our partners about this. Because it's very shameful for us. Or our partners can judge us. Please let us know, how we can convince them.".* To add to the context, a bisexual cisgender man expressed [excerpts], *"I would feel a little uncomfortable or rather unconfident if my partner prefers a toy over myself.".*

Four participants noted the initial difficulty in using sex toys and the time taken to understand how to use them. One participant expressed concerns related to the misuse of sex toys, four participants expressed related to sexual hygiene, and three responses described the fear of possible addiction or an increase in sensitivity/orgasm threshold. A heterosexual cisgender woman shared, "*I never had sex toys so I used jet sprays, hence the amount of pleasure that I get reduced each time while using so I had to use more, like for e.g. (drinking more alcohol to get the 'kick' each time). Therefore, I'm scared* [whether] *I could enjoy sex/sexual life with my partner in the future.*".

Strengths and Limitations of the Study

Sex toy use is widespread and linked to sexual well-being in English-speaking countries, but empirical data from other nations are lacking. The significant strength of the study lies in its attempt to bridge this gap in the literature. Furthermore, we aimed to overcome language barriers by circulating our questionnaire in 12 Indian languages, ensuring accessibility to diverse linguistic communities across the country rather than restricting the study to English users. In an Indian context, this study emphasizes the need to promote the responsible and safe use of sex toys, increase awareness about sexual hygiene, ensure proper usage, and ensure quality standards. Additionally, the mixed-method study design with an attempt to explore the qualitative aspects provided a fair picture of the existing issues regarding the acceptance, accessibility, availability, and anathemas prevalent in Indian society.

Acknowledging the study's limitations, such as its cross-sectional design, inability to prevent double entries, and online platform snow-ball sampling, it may not be fully representative of the entire Indian population, hence emphasizing the need for cautious interpretation. We tried to reduce the associated biases by recruiting a large sample size (oversampling).

Future research can focus on conducting studies with random sampling to obtain more generalizable results. Similarly, research on adolescent and elder populations, assessment for socioeconomic factors, the role of hygiene in sex toy use, longitudinal studies to explore changes in attitudes and behaviour related to sex toy use over time, etc. is required. Additionally, qualitative research could further investigate the experiences and opinions of individuals with diverse gender identities and sexual orientations and those from varied socioeconomic backgrounds to understand their unique perspectives on sex toy use.

Conclusion

This study contributes valuable insights into the proportion and experiences of sex toy use among Indian adults. Respondents shared diverse experiences; where majority of them reported positive experiences such as a role in understanding orgasms, enhanced sexual autonomy, better communication with partners, and enriched sexual experiences. On the other hand, concerns about social stigma, accessibility, affordability, privacy, awareness, and relational dynamics still exist. Negative responses related to physical discomfort and mental insecurities were also reported, though not as frequent. The qualitative analysis highlights a complex interplay between personal, relational, and societal factors influencing the use of sex toys, with barriers still prevalent within the Indian context.

References

1. Döring N, Poeschl S. Experiences with Diverse Sex Toys Among German Heterosexual Adults: Findings From a National Online Survey. *J Sex Res* 2020; **57(7):** 885–896. doi: 10.1080/00224499.2019.1578329

Questionnaire (English Version)

1. State

Andaman & Nicobar (UT)/ Andhra Pradesh/ Arunachal Pradesh/ Assam/ Bihar/ Chandigarh (UT)/ Chhattisgarh/ Dadra & Nagar Haveli and Daman and Diu (UT)/ Delhi / The Government of NCT and Delhi (UT)/ Goa/ Gujarat/ Haryana/ Himachal Pradesh/ Jammu & Kashmir (UT)/ Jharkhand/ Karnataka/ Kerala/ Ladakh (UT)/ Lakshadweep (UT)/ Madhya Pradesh/ Maharashtra/ Manipur/ Meghalaya/ Mizoram/ Nagaland/ Odisha/ Puducherry (UT)/ Punjab/ Rajasthan/ Sikkim/ Tamil Nadu/ Telangana/ Tripura/ Uttar Pradesh/ Uttarakhand/ West Bengal

2. Age group

☐ 18-24 ☐ 25-44

3. Sex

☐ Male ☐ Female ☐ Intersex

4. Gender

☐ Cisgender Man ☐ Cisgender Woman ☐ Transgender Man ☐ Transgender Woman ☐ Gender Non-binary ☐ Gender Non-conforming
☐ Genderqueer ☐ Agender ☐ Gender Fluid ☐ Other: _____________

5. Sexual orientation

☐ Heterosexual (straight) ☐ Gay (Homosexual man) ☐ Lesbian (Homosexual woman) ☐ Bisexual (+ spectrum)
☐ Queer ☐ Asexual (+ace spectrum) ☐ Pansexual ☐ Omnisexual ☐ Androsexual
☐ Gynesexual ☐ Autosexual ☐ Other: _____________

6. Do you have/ had sexual partner(s)?

☐ Yes ☐ No

What are sex toys?
Sex toys are sexual enhancement products with the intent of improving the nature and quality of sexual experiences. E.g. Dildos, vibrators, anal plugs, etc.

7. Did you know about/ Have you ever heard about sex toys before this?

☐ Yes ☐ No

8. Have you ever used a sex toy for SOLO sex (masturbation)?

☐ Yes ☐ No

9. Have you ever used a sex toy for PARTNERED sex?

☐ Yes ☐ No ☐ N/A (Never had a sexual partner)

For the following questions, use the following images for reference if you don't know what various toys mean

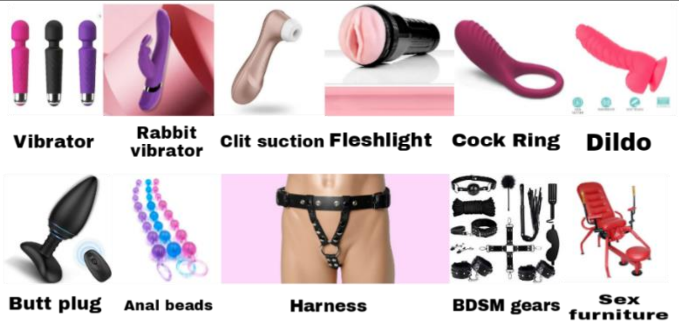

10. Which of the following sex toys have you used for SOLO sex (masturbation)?

Vibrators ☐ Yes ☐ No

Rabbit Vibrators	☐ Yes	☐ No
Clit suction toys	☐ Yes	☐ No
Dildos	☐ Yes	☐ No
Masturbators/ Fleshlights	☐ Yes	☐ No
Cock rings and similar	☐ Yes	☐ No
Butt plugs	☐ Yes	☐ No
Anal beads	☐ Yes	☐ No
Harnesses	☐ Yes	☐ No
BDSM gear	☐ Yes	☐ No
Sex furniture	☐ Yes	☐ No

11. Which of the following sex toys have you used for PARTNERED sex?

Vibrators	☐ Yes	☐ No	☐ N/A (Never had a sexual partner)
Rabbit Vibrators	☐ Yes	☐ No	☐ N/A (Never had a sexual partner)
Clit suction toys	☐ Yes	☐ No	☐ N/A (Never had a sexual partner)
Dildos	☐ Yes	☐ No	☐ N/A (Never had a sexual partner)
Masturbators/ Fleshlights	☐ Yes	☐ No	☐ N/A (Never had a sexual partner)
Cock rings and similar	☐ Yes	☐ No	☐ N/A (Never had a sexual partner)
Butt plugs	☐ Yes	☐ No	☐ N/A (Never had a sexual partner)
Anal beads	☐ Yes	☐ No	☐ N/A (Never had a sexual partner)
Harnesses	☐ Yes	☐ No	☐ N/A (Never had a sexual partner)
BDSM gear	☐ Yes	☐ No	☐ N/A (Never had a sexual partner)
Sex furniture	☐ Yes	☐ No	☐ N/A (Never had a sexual partner)

12. How would you rate general sexual satisfaction in life?

☐ (1) Very Dissatisfied
☐ (2) Dissatisfied
☐ (3) Neutral
☐ (4) Satisfied
☐ (5) Very Satisfied

13. Did you feel uncomfortable/ unconfident/ jealous if your partner wants to use a sex toy in partnered sex?

☐ Yes ☐ No ☐ N/A (Partner never wanted to use/ doesn't use sex toy or Never had a sexual partner)

14. Describe any OTHER positive and negative experiences (other than better or worse sexual experience) regarding use of sex toys. (Non-compulsory)

____
____
____
